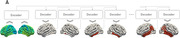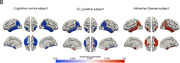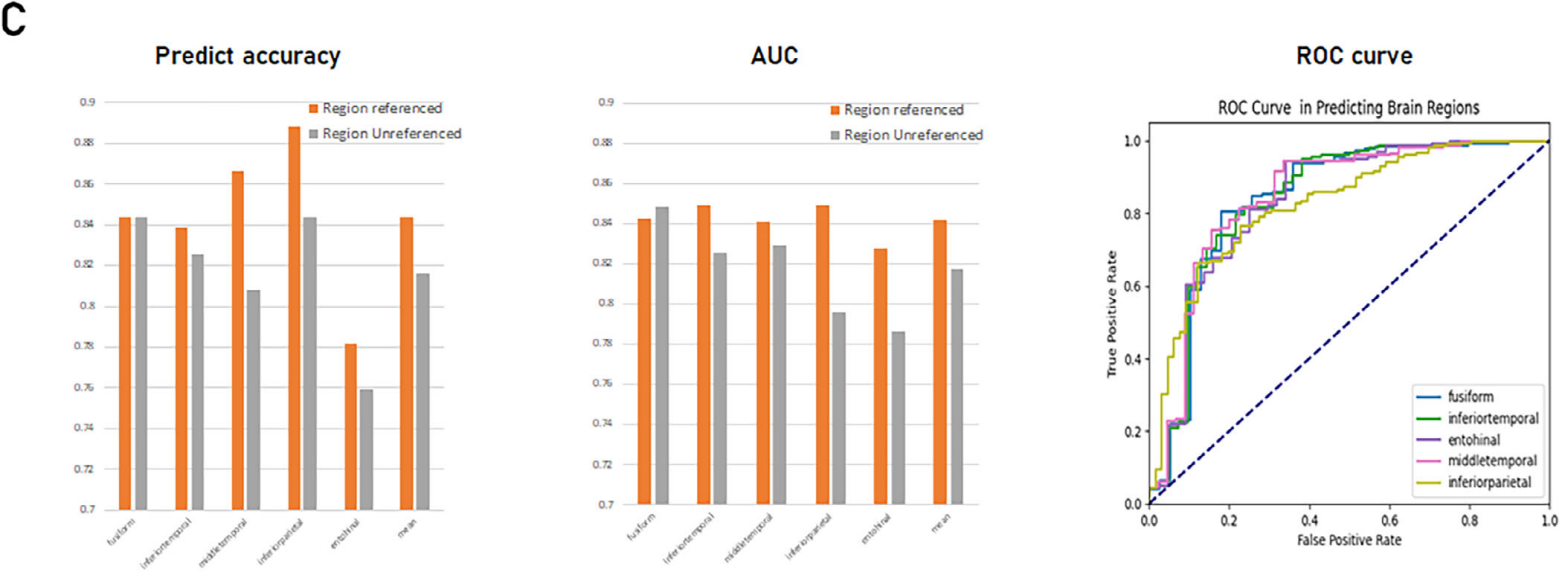# Attention mechanism‐based prediction of early tau accumulation using MRI

**DOI:** 10.1002/alz.092140

**Published:** 2025-01-03

**Authors:** Jin‐Yang Kim, Yeong‐Hun Song, Wha Jin Lee, Joon‐Kyung Seong, ADNI

**Affiliations:** ^1^ Korea University, Seoul Korea, Republic of (South); ^2^ NeuroXT, Seoul Korea, Republic of (South); ^3^ Alzheimer’s Disease Neuroimaging Initiative, http://adni.loni.usc.edu/, CA USA

## Abstract

**Background:**

Assessing tau accumulation in early affected areas like the lateral entorhinal cortex (EC) and inferior temporal gyrus (ITG) enables early prediction of disease progression and cognitive decline. However, positron emission tomography (PET) imaging poses radiation exposure and cost concerns. This research aims to develop a deep learning model predicting tau positivity in these regions using MRI.

**Method:**

In this study, we used the Alzheimer’s Disease Neuroimaging Initiative (ADNI) cohort, of which dataset was partitioned into train, validation, and test sets (8:1:1 ratio), encompassing a total of 1010 scans, all of whom underwent T1‐weighted magnetic resonance imaging (MRI) and [18F] flortaucipir‐PET imaging. For the T1‐weighted MRI images, FreeSurfer v7.2 was employed to perform pre‐processing and extract cortical thickness measurements. Simultaneously, [18F] flortaucipir‐PET imaging was processed to compute voxel‐wise regions of interest (ROIs) for 66 specific brain regions. Regional tau positivity was established using a cutoff at a z‐score of 1.25, with a focus on cognitive normal (CN) subjects within the train set. To predict early tau accumulation regions, we developed an attention mechanism‐based encoder‐decoder model by adopting a Transformer model into our problem setting, performing sequential predictions for each of the 66 regions. Notably, the model’s predictive performance in initial regions significantly influences subsequent predictions. Consequently, we implemented a prioritization strategy, emphasizing predictions from areas where the model demonstrated high accuracy. This approach was designed to enhance the overall predictive accuracy of the model.

**Result:**

Predicting five early tau accumulation regions per hemisphere, our model achieved an average AUC of 0.84 and accuracy of 84% for the test dataset (112 participants). Notably, in critical early disease progression regions (fusiform gyrus and ITG), AUC values of 0.84, 0.85, and accuracies of 84.4%, 84% were observed. Furthermore, the proposed prioritization strategy improved performance compared to predictions using vanilla attention‐based model.

**Conclusion:**

We developed an attention mechanism‐based architecture with an encoder‐decoder structure. By predicting outcomes not only based on cortical thickness values but also their cross‐attention‐based contexture information, we could achieve highly accurate tau prediction in early and challenging regions